# An Enhanced Starfish Optimization Algorithm via Joint Strategy and Its Application in Ultra-Wideband Indoor Positioning

**DOI:** 10.3390/biomimetics10050338

**Published:** 2025-05-20

**Authors:** Yu Liu, Maosheng Fu, Zhengyu Liu, Huaiqing Liu, Wei Peng, Ling Li, Yang Yang, Xiancun Zhou, Chaochuan Jia

**Affiliations:** School of Electronics and Information Engineering, West Anhui University, Lu’an 237012, China

**Keywords:** sine chaotic mapping, t-distribution, logarithmic spiral reverse, metaheuristic

## Abstract

The starfish optimization algorithm (SFOA) is a metaheuristic evolutionary intelligence algorithm with a great global search capability and strong adaptability. Although the SFOA has a good global search capability, it is not accurate enough in local search and converges slowly. To further enhance this convergence ability and global optimization ability, an enhanced starfish optimization algorithm (SFOAL) is proposed that combines sine chaotic mapping, *t*-distribution mutation, and logarithmic spiral reverse learning. The SFOAL can remarkably enhance both the global and local convergence capabilities of the algorithm, leading to a more rapid convergence speed and greater stability. In total, 23 benchmark functions and CEC2021 were used to test the development, search, and convergence capabilities of the SFOAL. The SFOAL was compared in detail with other algorithms. The experimental results demonstrated that the overall performance of the SFOAL was better than that of other algorithms, and the joint strategy could effectively balance the development and search capabilities to obtain stronger global and local optimization capabilities. For solving practical problems, the SFOAL was used to optimize the back propagation (BP) neural network to solve the ultra-wideband line-of-sight positioning problem. The results showed that the SFOAL-BP neural network had a smaller average position error compared to the random BP neural network and the SFOA-BP neural network, so it can be used to solve practical application problems.

## 1. Introduction

In many subject areas, such as engineering, science, and economics, a large number of complex optimization problems have arisen, such as large-scale production scheduling, complex system control, and nonlinear function optimization [1,2,3,4,5]. These problems are highly nonlinear, multimodal, and constrained. Traditional optimization algorithms face many difficulties in dealing with these problems, such as their proneness to local optimal solutions and inefficiency in computation, which makes it difficult to meet actual needs. Therefore, a more effective optimization method is needed. People have observed and studied phenomena such as biological evolution and group behavior in nature, finding that organisms have formed efficient adaptation and optimization mechanisms in the long-term evolution process [6,7,8,9,10]. For example, the inheritance, variation, and natural selection mechanisms of organisms enable species to continuously adapt to environmental changes and evolve in a better direction; for example, ant colonies can find the optimal path from the ant nest to a food source by transmitting pheromones during foraging, bird flocks can achieve the optimal group flight posture through cooperation and information sharing between individuals during flight, etc. [11,12,13]. These natural phenomena provide a rich source of inspiration for designing intelligent optimization algorithms.

Swarm intelligence algorithms have developed rapidly since the beginning of the 21st century [14,15,16,17]. On one hand, different intelligent optimization algorithms are integrated with each other, for instance, combining the genetic algorithm (GA) with the particle swarm optimization algorithm (PSO) or the ant colony algorithm with others. This integration capitalizes on their respective strengths, yielding more potent hybrid optimization algorithms [18,19]. Pan et al. developed an agent-assisted hybrid optimization (SAHO) algorithm to tackle the computationally expensive optimization problem [20]. SAHO combines teaching–learning-based optimization and differential evolution, obtaining stronger solving capabilities. Sangeetha et al. introduced a sentiment analysis method called the Taylor–Harris hawk optimization-driven long short-term memory network (THHO-BiLSTM) [21]. THHO-BiLSTM was formed by incorporating Taylor series into Harris hawk optimization (HHO), which can improve the performance of BiLSTM classifiers by selecting the optimal weights for hidden layers. Yıldız et al. used a new hybrid optimizer, AOA-NM (Arithmetic Optimization–Naider–Mead), to solve engineering design and manufacturing problems [22]. To address the AOA’s tendency to become trapped in local optima and boost solution quality, they incorporated the Naider–Mead local search method into AOA’s basic framework. This hybrid approach optimized AOA’s exploration and exploitation during the search.

On the other hand, these algorithms are combined with technologies in other fields, such as applying intelligent optimization algorithms to model selection and hyperparameter optimization in machine learning, as well as combining them with deep learning, big data processing, and other technologies, promoting the development of related fields [23,24,25]. Zheng et al. put forward a path prediction model integrating the GA, ant colony algorithm (ACO), and BP neural network, named GA-ACO-BP [26]. This model first conducts in-depth preprocessing on the original AIS data. It takes the BP neural network as the core prediction model. Leveraging the complementary nature of the GA and ACO, the GA determines the initial pheromone concentration of the ant colony. This effectively improves the convergence speed and performance of the traditional BP neural network. Cheng et al. proposed an approach for monitoring tool wear, which optimized the BP neural network with the firefly algorithm (FA) to enhance the accuracy of online tool wear prediction [27]. It did so by using the FA to modify the weights and thresholds of the BP neural network, which improved its performance. The experimental results validated the accuracy and reliability of this method.

In addition, new intelligent optimization algorithms continue to emerge, such as the artificial bee colony algorithm, bat algorithm, firefly algorithm, moth-to-fire optimization algorithm, etc., which further enrich the system of intelligent optimization algorithms [28,29,30]. The starfish optimization algorithm (SFOA) is an intelligent optimization algorithm developed based on the foraging behavior of starfish [31]. It aims to solve various optimization problems, especially complex problems difficult to handle with traditional methods. The SFOA simulates the foraging activities of starfish in a large range, allowing the algorithm to conduct a more extensive search in the entire solution space, increasing the chance of finding the global optimal solution and avoiding falling into the local optimum. During the search process, the movement strategy of individual starfish can be adaptively adjusted according to the search situation. For example, when approaching the optimal solution area, the step size may automatically decrease for a more refined search; in the early stages of the search, the step size is better able to quickly explore different areas. Although the starfish algorithm performs well, it still has problems, such as an insufficient local search capability and a slow convergence speed, and its global search capability needs to be improved. Therefore, this study modifies the SFOA through the joint strategy of sine chaotic mapping, t-distribution variation, and logarithmic spiral reverse learning to obtain an enhanced starfish optimization algorithm (SFOAL). It is found that the SFOAL has stronger local search and global search capabilities, as well as a faster convergence speed and greater stability, than the SFOA.

The subsequent parts of this study are arranged as follows: in Section 2, the principles underlying the SFOA and SFOAL are expounded upon in detail. In Section 3, the algorithms are rigorously evaluated, and the experimental results are dissected meticulously. In Section 4, the practical implications of the algorithms for engineering problems are explored. Finally, a summary of the key findings is presented, encapsulating the main outcomes of this study. Additionally, suggestions for future research directions are put forward, highlighting potential areas for further exploration and improvement.

## 2. SFOA

The exploration, predation, and regeneration behaviors of starfish inspired the SFOA, which also has its own exploration and development phases. Most popular algorithms use vector search mode in the exploration phase to process separable functions with good performances, but they are inefficient in processing inseparable functions. One-dimensional search mode is efficient in processing inseparable functions but may fall into local convergence or converge slowly. During the exploration stage, the SFOA capitalizes on the five-armed structure of the starfish (where eyes are located on the arms) to integrate five-dimensional and one-dimensional search strategies. Depending on the dimension *d*, it employs the following distinct search patterns: a five-dimensional search when *d* > 5 and a one-dimensional search when *d* ≤ 5. This approach is designed to address the previously mentioned drawbacks. In the development stage, the SFOA is designed via predation and regeneration strategies. The predation strategy is the main update process, and a parallel bidirectional search using the information of the two starfishes is used to encourage the candidate solution to move to a better position.

## 3. SFOAL

The SFOAL modifies the SFOA by introducing a multi-strategy mode of sine chaotic map initialization population, t-distribution mutation, and logarithmic spiral reverse learning to update the position. By introducing multiple strategies, the search space can be explored from different angles, increasing the possibility of the algorithm finding the optimal solution in the global scope. When the algorithm uses a single strategy, it is easy to fall into the local optimal solution and be unable to jump out, with the final result not being the global optimal. Multi-strategy modification can provide the algorithm with more opportunities to jump out of the local optimal solution by switching between different strategies.

### 3.1. Initialization with Sine Chaotic Map

Initial conditions have a significant impact on the sine chaotic map. Even the slightest change in these initial conditions may result in chaotic sequences that are completely different from one another. This feature can be used to initialize multiple different populations or search directions in intelligent optimization algorithms, thereby increasing the diversity of the algorithm. For complex multi-peak optimization problems, different initial conditions allow the algorithm to start searching from different starting points, increasing the probability of finding the global optimal solution. In the initialization stage, the positions of the starfish are randomly produced. *x*_ij_ denotes the position of the *i*th starfish within the *j*th dimension and is expressed in matrix form. The size of the matrix is n×d, where *n* represents the population size and *d* represents the dimension of the design variable.

Sine chaotic mapping is a classic one-dimensional mapping method, which the SFOAL uses to optimize the initial population of starfish. Its mathematical expression is shown in Equation (1), as follows:

(1) $$x_{n+1}=\frac{\delta \sin(\pi x_{n})}{4(ub_{j}-lb_{j})}+lb_{j}$$

where *δ* is a constant between 0 and 4. When it is greater than 3.8, the mapping system shows a chaotic state, and the closer it is to 4, the more obvious this chaotic state is. Here, *δ* is equal to four. *ub*_j_ and *lb*_j_ are the upper and lower boundaries of the variables, respectively. *x*_n_ and *x*_n+1_ represent the current position of the starfish and its position after sine chaotic mapping, respectively.

After the position is initialized, the positions of all starfish are evaluated to obtain the fitness value of each starfish, which are saved and updated using the vector ***F*** of size n × 1, as seen in Equation (2), as follows:

(2) $$F={\left[\begin{array}{c} F_{1}\\ F_{2}\\ \ldots \\ F_{n} \end{array}\right]}_{n\times 1}$$

### 3.2. Exploration

After completing the position initialization, the size of *G*_p_ will be compared to a number in the range (0, 1); when the number is not greater than *G*_p_, the exploration phase will begin. Then, *d* is determined, and if *d* is greater than five, the starfish moves its five arms to explore the environment. Moreover, the starfish’s movement is updated by searching for the best position information in the agent. The model for this stage is presented in Equation (3), as follows:

(3) $$\left\{\begin{array}{cc} P_{i,p}^{t}=x_{i,p}^{t}+a_{1}(x_{best,p}^{t}-x_{i,p}^{t})\cos\theta , & rand<0.5\\ P_{i,p}^{t}=x_{i,p}^{t}+a_{1}(x_{best,p}^{t}-x_{i,p}^{t})\sin\theta , & rand>0.5 \end{array}\right.$$

where $P_{i,p}^{t}$ denotes the obtained position; $x_{i,p}^{t}$ and $x_{best,p}^{t}$ denote the current position and current best position of dimension *p*, respectively; and *p* denotes five randomly selected dimensions of dimension *d*. *a*_1_ and *θ* are obtained using Equations (4) and (5), respectively, as follows:

(4) $$a_{1}=(2r-1)\pi$$

(5) $$\theta =\frac{\pi }{2}\cdot \frac{t}{t_{\max}}$$

where *t* and *t*_max_ represent the present and maximum iteration number, respectively. During the exploration phase, *a*_1_ is randomly generated and used to update the position, while *θ* varies with the number of iterations. These two parameters jointly assess the influence of the distance between the optimal and present position in the chosen update dimension. For the position outside the boundary after the update, the previous position is maintained instead of moving to the updated position, as shown in Equation (6), as follows:

(6) $$x_{i,p}^{t+1}=\left\{\begin{array}{cc} P_{i,p}^{t} & l_{b}\leq P_{i,p}^{t}\leq u_{b}\\ x_{i,p}^{t} & else \end{array}\right.$$

where *p* denotes the update dimension, and *l*_b_ and *u*_b_ denote the lower and upper bounds of the design variables, respectively.

If d≤5, one arm of the starfish will move to search for food sources while using the position information of the other starfish. The formula for the position is shown in Equation (7), as follows:

(7) $$P_{i,q}^{t}=E_{t}x_{i,p}^{t}+b_{1}\left(x_{k_{1},p}^{t}-x_{i,p}^{t}\right)+b_{2}\left(x_{k_{2},p}^{t}-x_{i,p}^{t}\right)$$

where $x_{k_{1},p}^{T}$ and $x_{k_{2},p}^{T}$ are the dimensional positions of two selected starfish, *b*_1_ and *b*_2_ are two numbers in the range (1, −1), and *p* is a number randomly chosen in the *d* dimension. *E*_t_ is the energy of the starfish, obtained via Equation (8), as follows:

(8) $$E_{t}=\frac{t_{\max}-t}{t_{\max}}\cos\theta$$

*θ* is calculated via Equation (5), and the position out of the bounds is determined in the same way as before.

### 3.3. Exploitation

During the development phase, two update strategies were designed for the SFOA. The SFOA uses a parallel bidirectional search strategy that requires the use of information from other starfish and the present optimal position in the population. First, five distances between the optimal position and other starfish are calculated, then two distances are randomly selected as confirmation information, and the position of each starfish is updated using a parallel bidirectional search strategy. The distance can be obtained via Equation (9), as follows:

(9) $$d_{o}=\left(x_{best}^{t}-x_{o_{p}}^{t}\right),o=1,2,3,4,5$$

where *d*_o_ is the distance between the best position and other starfish, and *o*_p_ is the five randomly chosen starfish.

Therefore, the position update rule for each starfish for the predation behavior can be obtained via Equation (10), as follows:

(10) $$P_{i}^{t}=x_{i}^{t}+r_{1}d_{o1}+r_{2}d_{o2}$$

where *r*_1_ and *r*_2_ are random numbers between 0 and 1, and *d*_o1_ and d_o2_ are randomly selected from *d*_o_.

In addition, starfish are vulnerable to attacks from other predators during the predation process. When a predator attempts to catch a starfish, the starfish might sever and jettison one of its arms to elude capture. In the starfish optimization algorithm, this concept is reflected in the regeneration phase, which is carried out solely on the last starfish within the population. The formula for updating the position during this regeneration phase is presented in Equation (11), as follows:

(11) $$P_{i}^{t}=\exp(-t\times n/t_{max})x_{i}^{t}$$

For out-of-bounds positions, the following update rules are used (Equation (12)):

(12) $$x_{i}^{t+1}=\left\{\begin{array}{ccc} P_{i}^{t} & & l_{b}\leq P_{i}^{t}\leq u_{b}\\ l_{b} & & P_{i}^{t}<l_{b}\\ u_{b} & & P_{i}^{t}>u_{b} \end{array}\right.$$

### 3.4. T-Distribution Mutation

The basic aim of updating the population position using t-distribution mutation is to introduce mutation by using the characteristics of t-distribution, which brings a certain degree of randomness and robustness to the search process. After each position update of the starfish, the SFOAL incorporates t-distribution variation. This addition accelerates the convergence rate of the SFOA and enhances its stability and accuracy. The position update formula is established in Equations (13) and (14), as follows:

(13) $$P_{n}^{t}=P_{best}^{t}+P_{best}^{t}\cdot t(iter)$$

(14) $$x_{i,p}^{t+1}=\left\{\begin{array}{cc} P_{n}^{t} & F_{new}<F_{i}\\ x_{i,p}^{t+1} & else \end{array}\right.$$

where $P_{n}^{t}$ represents the new position after the t-distribution mutation disturbance, $P_{best}^{t}$ represents the best position searched by the current discoverer, t(iter) represents the *t*-distribution in which the degree of freedom is given by the present iteration number, $x_{i,p}^{t+1}$ represents the position of the *i*th starfish, *F*_new_ represents the fitness value of the new position, and *F*_i_ represents the fitness value of the *i*th starfish. When *F*_new_ < *F*_i_, the new position obtained after the *t*-distribution mutation disturbance is better than the current starfish position, and the position is updated to the starfish.

### 3.5. Logarithmic Spiral Opposition-Based Learning

Logarithmic Spiral Opposition-Based Learning (LSOBL) focuses specifically on the reverse solution of the optimal individual within the boundary. As the iteration progresses, the position of the optimal individual constantly changes, and the spiral reverse learning search space of OBL decreases. However, LSOBL operates on individuals that change in a smaller space (especially those close to the best solution), which is very valuable in the later iterations when the population diversity decreases. This can prevent the algorithm from converging prematurely and ensure that the global optimal value is not missed. Moreover, the LSOBL model is defined in Equations (15) and (16), as follows:

(15) $$P_{op}^{t}=r_{1}\cdot ub_{j}+r_{2}\cdot lb_{j}-P_{best}^{t}$$

(16) $$x_{i,p}^{t+1}=\left|P_{best}^{t}-P_{op}^{t}\right|\times e^{bl}\times \cos(2\pi l)+P_{best}^{t}$$

**Figure 1:**
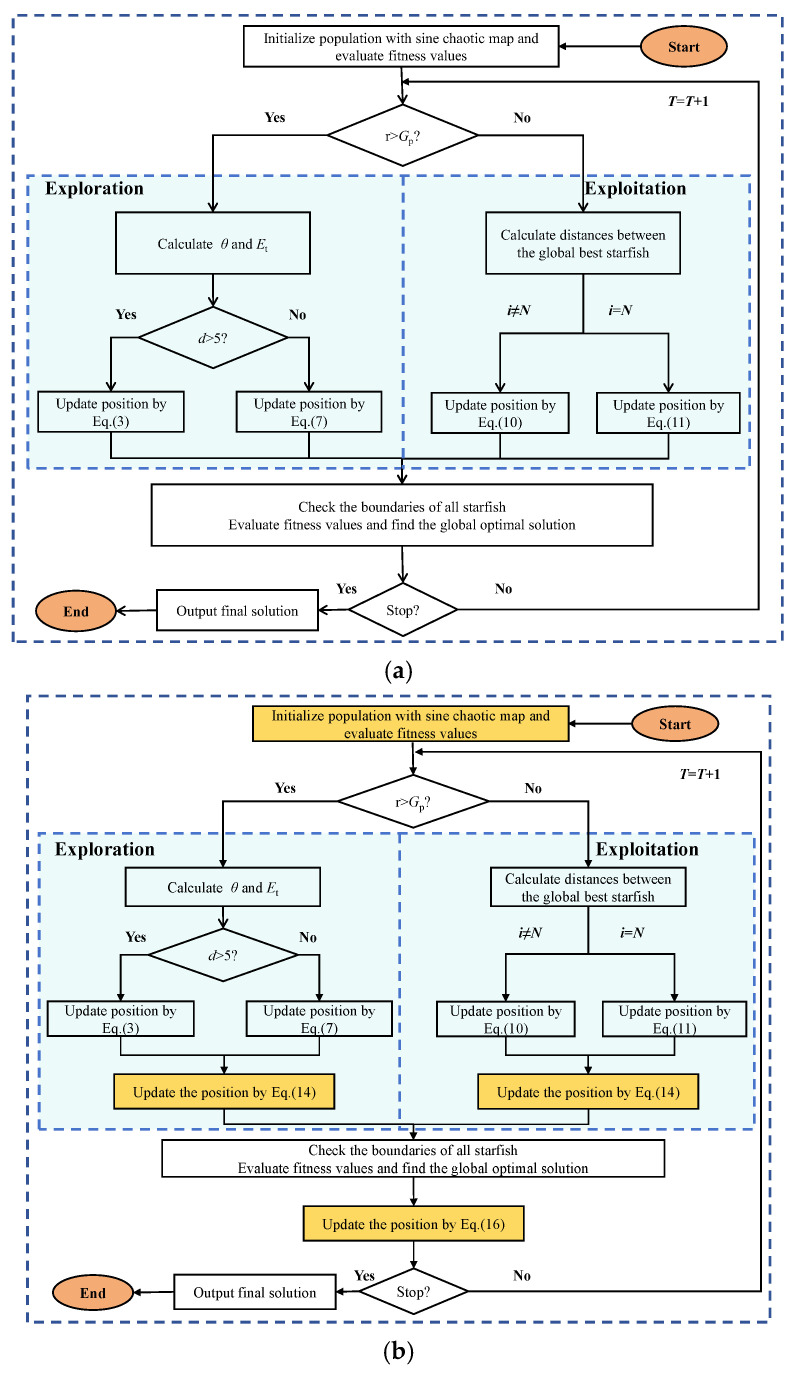
The flowcharts of SFOA and SFOAL. (**a**) SFOA flowchart and (**b**) SFOAL flowchart.

Among them, $P_{op}^{t}$ is the position of the spiral after reverse learning, and *l* is a random number between −1 and 1. Flowcharts of SFOA and SFOAL are presented in Figure 1.

## 4. Experimental Results and Analysis

### 4.1. Experimental Environment

The simulation platform is configured with Windows 11, a 12th-generation Core i7 processor with a 2.10 GHz frequency, and 16 GB of dynamic memory. All algorithms are realized in MATLAB R2023a.

### 4.2. Benchmark Functions

Section 4 describes the utilization of the benchmark functions to evaluate the performances of all the algorithms, including the SFOAL, SFOA, grey wolf optimizer (GWO) [32], goose algorithm (GOOSE) [33], GA [34], pied kingfisher optimizer (PKO) [35], dreaming optimization algorithm (DOA) [36], crayfish optimization algorithm (COA) [6], beluga whale optimization algorithm (BWO) [37], and escape algorithm (ESC) [38]. Functions *F*_1_ to *F*_7_ in Appendix A (Table A1) are single peak functions for verifying the convergence speed of the evolutionary algorithm. Functions *F*_8_ to *F*_23_ in Appendix A (Table A2 and Table A3) are multi-peak functions with multiple local optimal values that can test the ability to avoid premature convergence.

#### 4.2.1. Unimodal Functions

Table 1 lists the results of different methods on the unimodal benchmark functions in 20 independent runs. The statistics include the best fitness value (min), standard deviation (std), and mean fitness value (avg) of the function. Table 1 shows that for functions *F*_1_ to *F*_4_, the SFOAL and SFOA can find accurate optimal solutions. For functions *F*_5_ and *F*_6_, the SFOAL has the best optimal value, standard deviation, and average value. For function *F*_7_, the SFOAL ranks second in statistics, only being worse than the COA, but it is improved compared to the SFOA. Therefore, the SFOAL can effectively utilize the search space to produce satisfactory results for unimodal functions and has strong search capabilities.

#### 4.2.2. Multimodal Functions

Multimodal functions *F*_8_ to *F*_13_ are used to evaluate the exploration ability of the evolutionary algorithm, and their results are presented in Table 2. From the data in the table, we can see that for functions *F*_8_, *F*_12_, and *F*_13_, the SFOAL sees a significant improvement over the SFOA, with better optimal values and a smaller standard deviation. Among all algorithms, the SFOAL ranks in the top three. The SFOAL, which represents an improvement on the joint strategy, can effectively avoid premature convergence and falling into a local optimal solution. For functions *F*_9_ to *F*_11_, SFOAL ranks first and has a smaller standard deviation and better optimal values compared to other algorithms, showing that the SFOAL has a satisfactory ability to find the global optimal solution.

#### 4.2.3. Fixed Dimension Multimodal Functions

The results of multimodal functions (*F*_14_–*F*_23_) with fixed variables are presented in Table 3 and Table 4. As the table shows, for multimodal functions of fixed dimensions, the SFOAL shows an excellent global optimization ability and stability compared with other functions. For functions *F*_14_ to *F*_23_, the SFOAL has the smallest mean and a small standard deviation. This shows that by combining sine chaotic mapping, t-distribution variation, and the logarithmic spiral reverse learning strategy, it is possible to effectively avoid falling into local convergence and enhance the stability of the algorithm.

**Figure 2:**
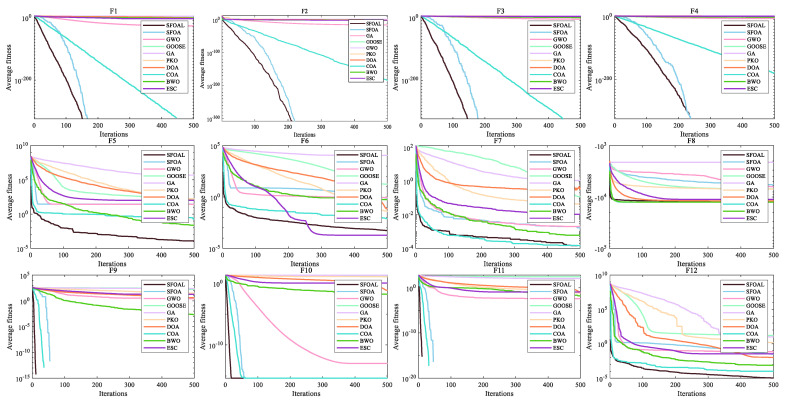

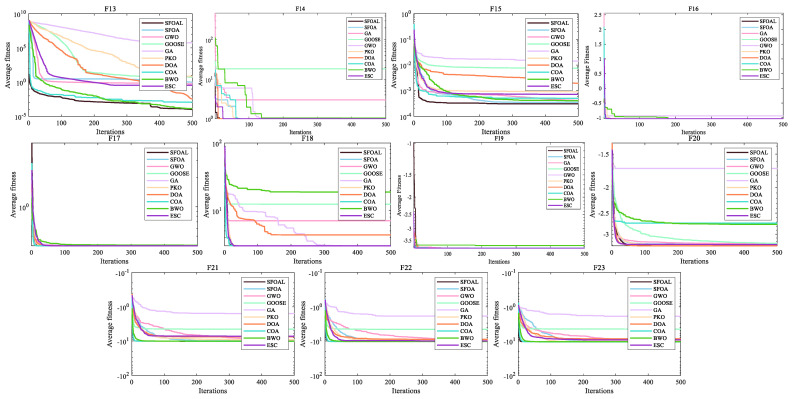
Convergence graphs of all functions.

Figure 2 shows the convergence curves of all functions. We can see that SFOAL has obvious advantages. For functions *F*_1_ to *F*_7_, compared with the SFOA, the convergence speed is greatly improved, indicating that the joint-strategy SFOAL can find the optimal solution of the unimodal function faster and has a strong search ability. For functions *F*_8_ to *F*_13_, the SFOAL is significantly improved compared with the SFOA, and it also has the fastest convergence speed compared with other algorithms. For *F*_14_ to *F*_23_, the SFOAL is significantly improved compared with the SFOA. The SFOAL also has a faster convergence speed and a better average fitness value than other algorithms. For the convergence curve analysis, the SFOAL modified using the joint strategy greatly improves the stability and global optimization ability of the SFOA, and it can obtain a faster convergence speed and a smaller fitness value.

**Figure 3:**
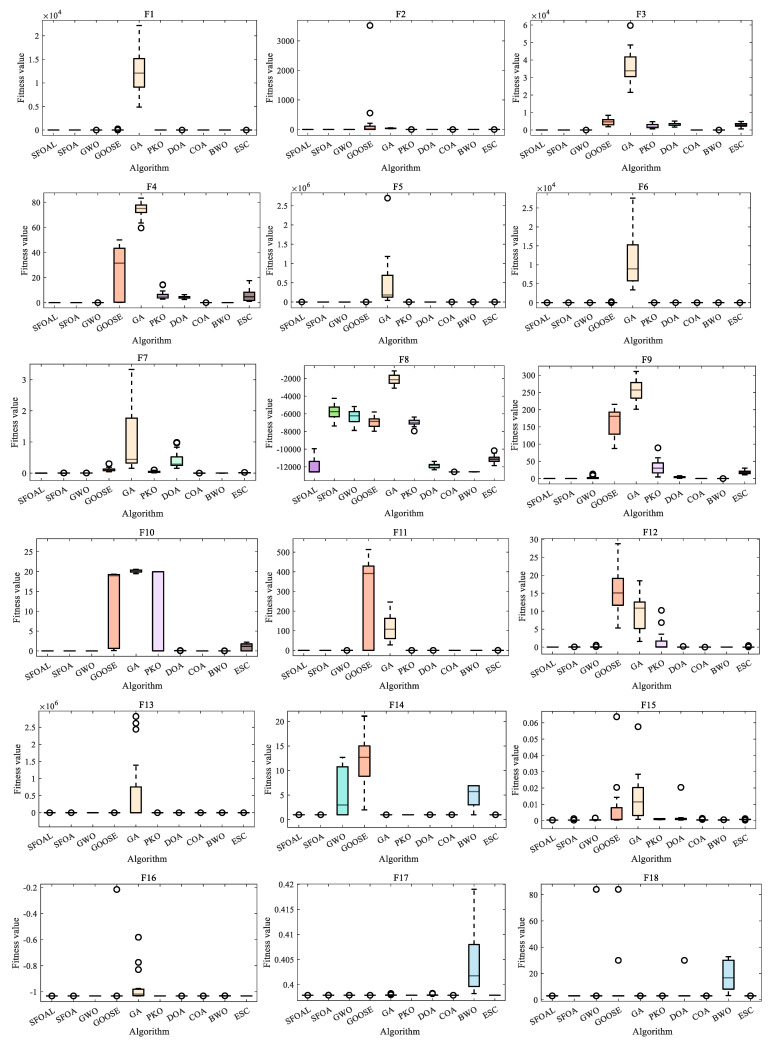

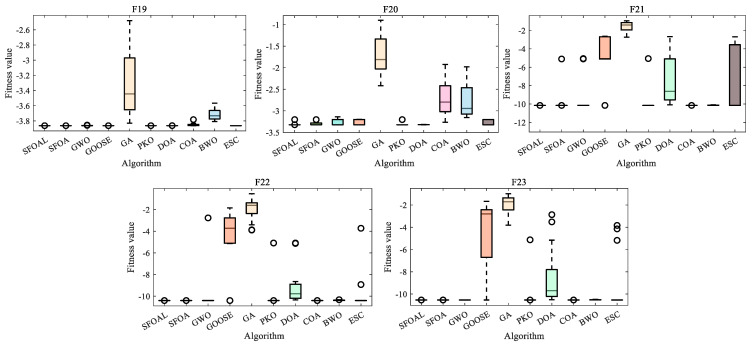
ANOVA test of all functions.

Figure 3 shows the ANOVA test of all functions. From the ANOVA test diagram, it can been seen that, for unimodal functions (*F*_1_–*F*_7_), the SFOAL shows lower fitness values and smaller fluctuations compared with other algorithms, indicating that it has certain advantages in terms of convergence speed, solution quality, and stability. For *F*_8_, the fitness value of the SFOAL is about −12,569, while the fitness value of the SFOA is about −7380, with the SFOAL being significantly lower than the SFOA. For *F*_9_ to *F*_13_, the fitness values of the SFOAL and SFOA are mostly zero or close to zero, and the two perform similarly, showing a good performance and the ability to quickly converge to a better solution or avoid falling into a poor solution area. Compared with other algorithms, the SFOAL performs better overall. The SFOAL can mostly maintain low fitness values on multiple functions, showing that it has a strong ability to find the optimal solution or a better solution, and it also has certain advantages in terms of stability. For *F*_15_ to *F*_23_, the SFOAL has a relatively stable overall performance under different fitness value evaluations. In most cases, the fitness value is low and the change range is small, indicating that it has a high stability. Compared with the SFOA, its fitness value is relatively close, and in most cases, the difference between the two values is not large. Compared with other algorithms, the SFOAL has a better optimization ability and stability. As mentioned above, the SFOAL modified by the joint strategy has a better stability and global optimization ability, and it can avoid falling into local convergence too early.

The results of Wilcoxon rank sum test are presented in Table 5. In statistics, 0.05 is usually used as the significance level threshold. If the *p* value is less than 0.05, there is a significant difference between the two algorithms. In the statistical results, there are significant differences between the SFOAL and the GWO, GOOSE, GA, PKO, and DOA for all 23 test functions, indicating that the performances of the SFOAL and these algorithms are very different. There are significant differences among the GOOSE, BWO, and ESC for 22 test functions, indicating that the SFOAL also has obvious performance differences from them. There are significant differences with GWO for 21 test functions, which also shows that the performance difference is quite significant. The number of significant differences with the SFOA and COA is relatively small, but differences are also shown for most test functions. In summary, there are significant differences between the SFOAL and the algorithms in the table for most test functions, which shows that the SFOAL has obvious performance differences from the other algorithms.

**Figure 4:**
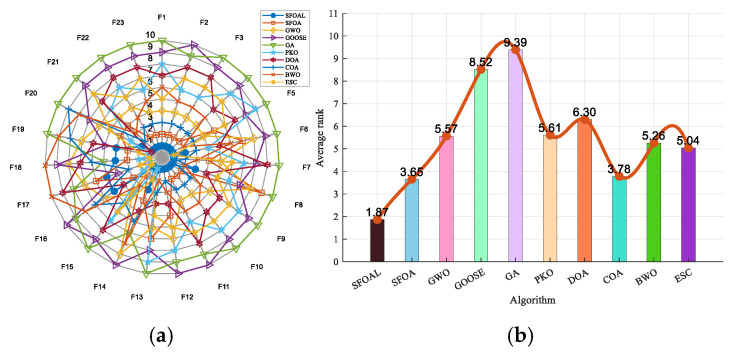
Radar and ranking charts for benchmark functions. (**a**) Radar chart and (**b**) ranking chart.

In the radar chart (Figure 4a), the distribution of the SFOAL (blue dots) on *F*_1_ to *F*_23_ is more concentrated in the inner circle, which shows that the SFOAL performs better on *F*_1_ to *F*_23_ and has a better performance than the other algorithms. From the ranking chart, it can be seen that the average ranking of the SFOAL is 1.87, which is the highest average ranking among all algorithms. This shows that, considering all evaluation functions (*F*_1_ to *F*_23_), the SFOAL has a clear advantage in overall performance and performs better than the other algorithms. In summary, for benchmark functions, using the joint strategy to improve the SFOA is a feasible solution. The SFOAL can improve the convergence speed and global optimization ability of the algorithm, obtain more stable results, and avoid falling into local convergence.

### 4.3. CEC2021 Test Functions

CEC2021 covers unimodal functions, multimodal functions, and composite functions, which can comprehensively evaluate the performances of optimization algorithms.

**Figure 5:**
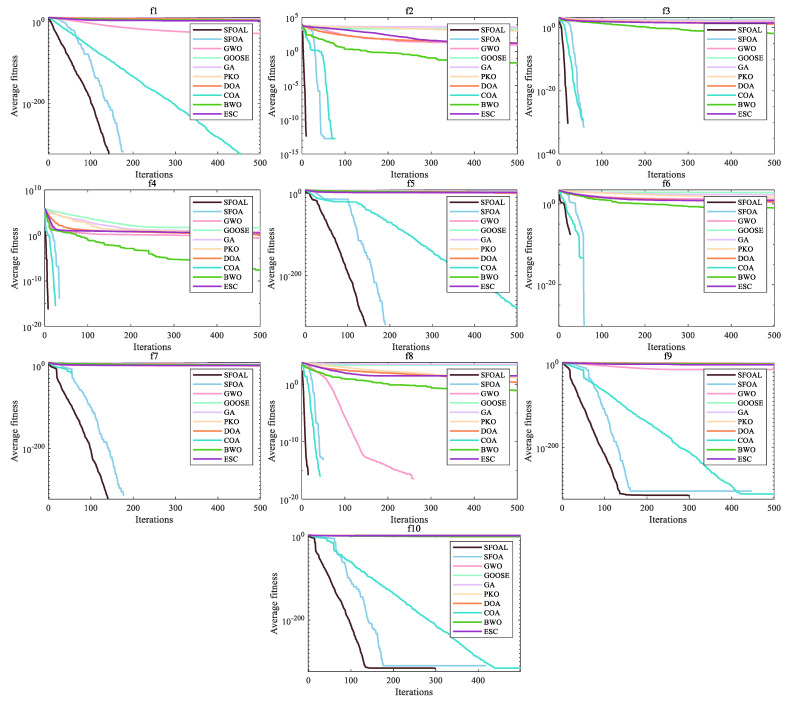
Convergence graphs of CEC2021 test functions.

Table 6 depicts the results for the CEC2021 test set. For the CEC2021 test set, the SFOA has a strong large-scale optimization ability and stability. The SFOAL modified via the joint strategy retains the global optimization ability and stability of the SFOA. Both algorithms can find accurate optimal solutions. Compared with other algorithms, the SFOAL has an absolute advantage. Convergence curves are shown in Figure 5. The figure shows that, although SFOA can obtain an accurate optimal solution, its convergence speed is worse than that of algorithms such as DBO. The SFOAL improved via the joint strategy shows an unmatched advantage over the other algorithms in terms of convergence speed. For functions *f*_1_–*f*_10_, the SFOAL can converge faster and accurately find the best solution for the function, showing its powerful global solution ability and avoiding premature convergence.

**Figure 6:**
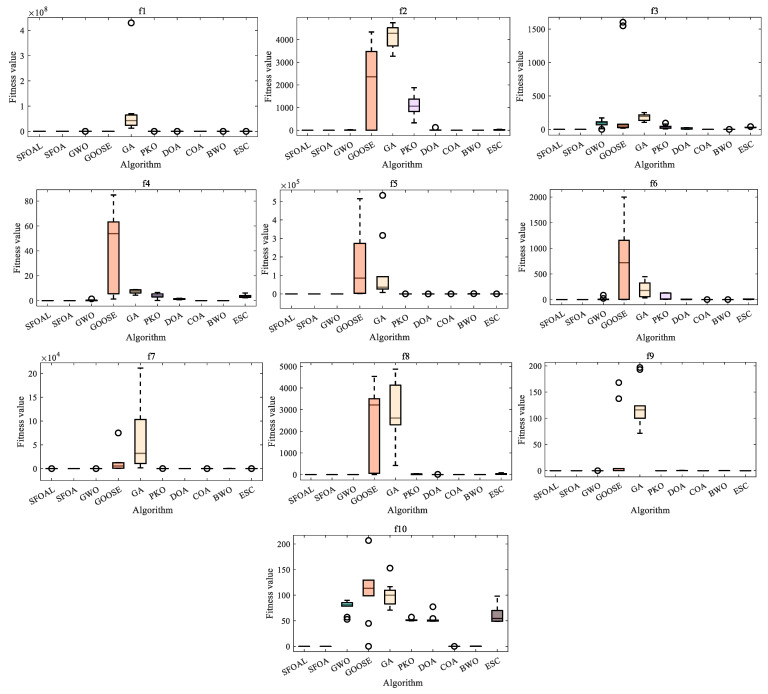
ANOVA test of CEC2021 test functions.

The ANOVA test results for CEC2021 are presented in Figure 6. The figure shows that for functions *f*_1_–*f*_10_, the SFOAL shows an extremely strong stability and can accurately find the best solution. Compared with other functions, only the original SFOA can compare to it in terms of stability, and other functions do not show an extremely strong stability on all functions. In summary, for the CEC2021 test set, the SFOAL after the joint strategy can maintain the stability and global optimization ability of the SFOA with a greatly improved convergence speed, indicating that the joint strategy can enhance the search ability of the algorithm.

## 5. Ultra-Wideband Indoor Localization

### 5.1. The Ultra-Wideband Localization Principle

**Figure 7:**
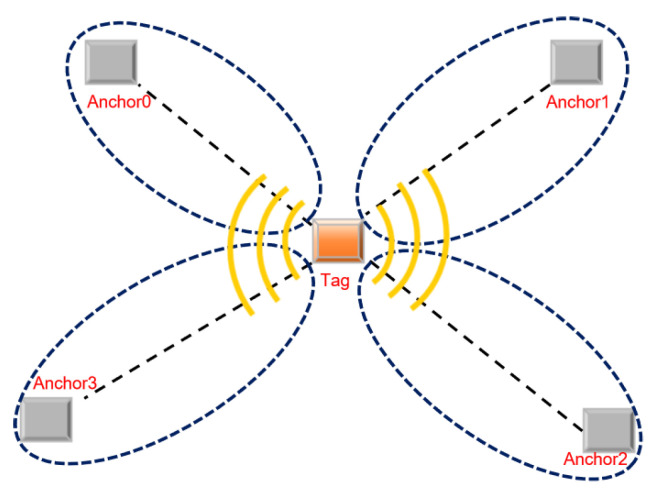
The diagram of the UWB positioning principle.

Ultra-wideband indoor positioning (UWB) is achieved by arranging four positioning base stations with known coordinates indoors, as shown in Figure 7. The personnel or equipment that need to be positioned carry a positioning tag. The tag sends pulses at a certain frequency and continuously measures the distance with the four base stations with known positions.

### 5.2. Back Propagation Neural Network

**Figure 8:**
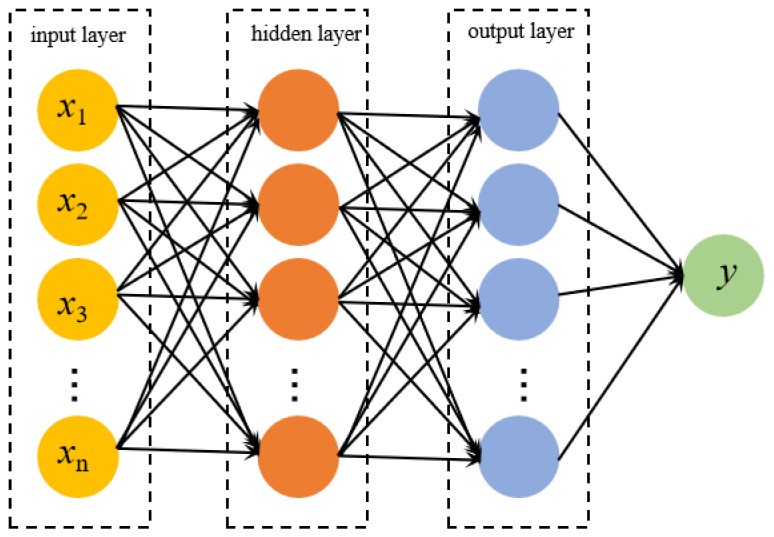
BP neural network structure diagram.

The BP neural network is a forward feedback neural network, which consists of an input layer, several hidden layers, and an output layer. The BP neural network includes the following two components: forward propagation and back propagation. During the forward propagation phase, input data initiate from the input layer. They successively undergo calculations in the hidden layers and ultimately arrive at the output layer. At each layer, a neuron conducts a weighted sum of the received input signals. Subsequently, it undertakes a nonlinear transformation via the activation function to generate an output, which is then transmitted to the next layer. In the back propagation phase, the error at the output layer is computed. This error represents the disparity between the predicted value and the actual value. Then, the error is backpropagated from the output layer to the input layer, and the connection weights between the neurons in each layer are adjusted according to the error so that it gradually decreases. Here, a simplified three-layer network model is utilized, as shown in Figure 8.

### 5.3. Hybrid Positioning Algorithm

The hybrid BP algorithm is a new method that combines the SFOA or SFOAL with the BP neural network. The algorithm combines the comprehensive and targeted exploration function of the starfish algorithm with the nonlinear fitting and approximation function of the BP neural network to enhance the optimization ability and prediction accuracy of the algorithm.

The algorithm follows the following steps:

1. Position initialization: The initialization method of the starfish algorithm is used to determine the starting position.
2. Input data determination: The starfish position is used as the input data for the BP neural network training and prediction.
3. BP neural network training: The difference between the expected output and the actual output is used as the loss function. Back propagation is used to adjust the weights and bias of the network to minimize the error.
4. Starfish position update: The position of the starfish is updated according to the update strategy of the starfish algorithm and the results of the BP network training.
5. Iterative execution: Steps 2 to 4 are repeated until the termination condition is met.

The hybrid algorithm effectively uses the global search capability to find the initial position of the optimal solution by combining the SFOA or SFOAL with the BP neural network, and it improves accuracy and precision through BP network training. This hybrid algorithm provides an excellent optimization and prediction performance for complex problems. Using hybrid algorithms for UWB positioning can improve positioning accuracy and stability.

**Figure 9:**
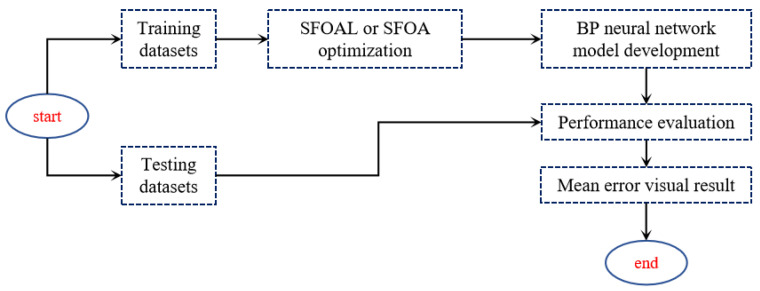
Flowchart of hybrid algorithm optimization BP neural network.

Figure 9 describes the evolution of the hybrid algorithm. First, the dataset is divided into a training set and a validation set. Subsequently, random values are assigned to the weights and thresholds of the BP neural network, and the training set is input into the network for training. The SFOA adopts the global optimal search solution through random and local search strategies. Finally, a BP neural network model optimized via the starfish algorithm is obtained. The test dataset is then input into the final model to predict its results. To measure the accuracy and reliability of the model, the predicted results are compared with the true coordinates, and the prediction error is calculated to evaluate the performance of the model. Through these steps, the hybrid BP model is trained, optimized, and evaluated, making it suitable for predicting and optimizing positioning data.

### 5.4. Line-of-Sight (LOS) Scenario

To verify the accuracy of UWB positioning and the effectiveness of the algorithm and provide necessary data for subsequent research, an experimental environment was built, systematic experiments were conducted, and the performance and practical feasibility of UWB positioning were evaluated. A line-of-sight (LOS) scenario was built here.

**Figure 10:**
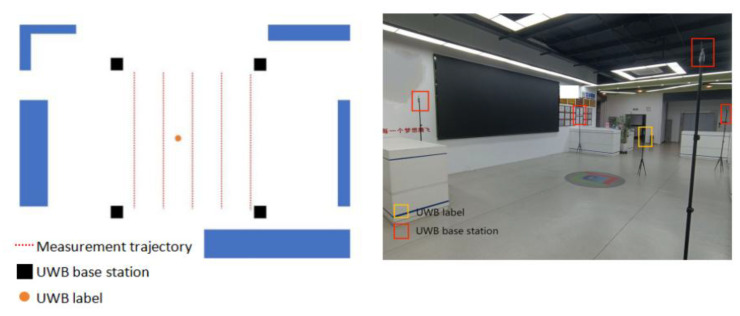
Actual test scene diagram.

To simulate the indoor positioning scenario, four UWB base stations were installed in the designated area, each with a height of 2.2 m and located at different coordinates, (0,0), (0,5), (5,0), and (5,5). These base stations were used to provide known location information for indoor positioning. The height of the tag was set to 1.8 m. In this environment, the UWB base station provided a stable reference system for determining the location of the target object. By cooperating with the upper-level computer software, the real-time location of the UWB tag could be obtained, thereby recording and analyzing the indoor positioning data. Figure 10 shows the experimental scenario.

**Figure 11:**
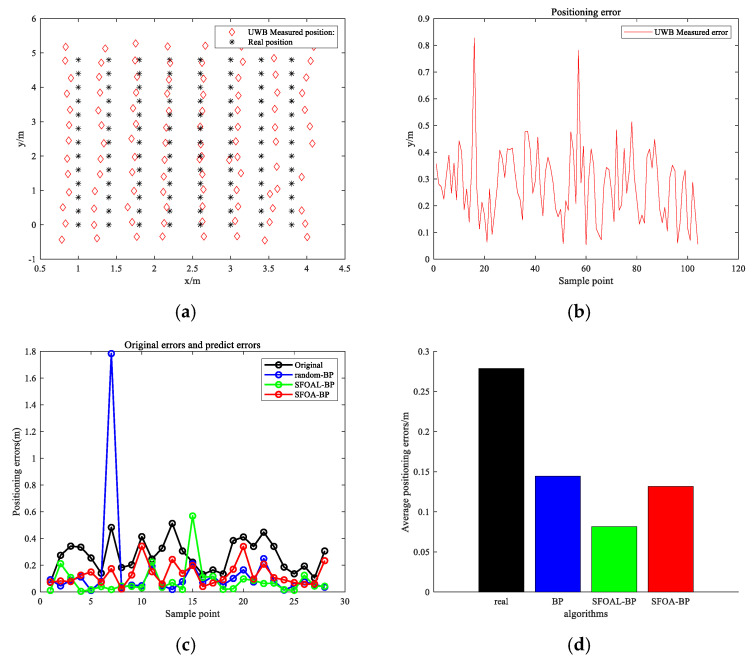
Line-of-sight positioning test results. (**a**) UWB measured position, (**b**) positioning error, (**c**) predict errors and, (**d**) average positioning errors.

Figure 11a,b show the test results of traditional UWB positioning technology. The black asterisk in Figure 11a is the actual position, and the red diamond is the UWB positioning measurement value. The figure shows that the overlap rate between the two is low, indicating that traditional UWB positioning technology has a large positioning error. First, we made a detailed comparison between the positioning measurement data and the actual coordinate position to confirm that the algorithm needed to be optimized to improve the positioning accuracy. As Figure 11b shows, there are obvious errors in the UWB positioning results. The positioning accuracy of some points does not reach the centimeter level. This phenomenon is very common in traditional UWB positioning technology, indicating that there are large fluctuations or deviations. To address this problem, the sample set was divided into a training dataset and a test dataset, and then the BP neural network model and the hybrid BP model were input for position prediction. Figure 11c,d show the test error and average position error of the sample points, respectively. As shown in Figure 11c, the blue line is the BP network test result, the green line is the SFOAL-BP hybrid model test result, and the red line is the SFOA-BP hybrid model test result. As the figure shows, for most sample points, the position error of the SFOAL-BP hybrid model achieves the minimum value, and only a few points have large errors. The error of SFOA-BP is greater than that of the SFOAL optimization model, but it has a greater advantage over the BP model. As shown in Figure 11d, the average position error of SFOAL-BP is 0.0814 m, which is the smallest among the three models, and the SFOA-BP model ranks second (the average position error is 0.0136 m). This shows that the SFOAL with joint strategy optimization can effectively jump out of the local optimal solution and obtain stronger global optimization capabilities.

## 6. Conclusions and Future Work

In summary, this study modifies the SFOA by combining sine chaotic mapping, t-distribution mutation, and the logarithmic spiral reverse learning strategy to obtain a strong global optimization ability and stability. A total of 23 basic functions, the CEC2021 function set, and an ultra-wideband indoor positioning problem are used to evaluate the development, exploration, and convergence ability of the SFOAL. Through testing the unimodal function, it is found that the joint strategy can enhance the local optimization ability of the algorithm. The SFOAL has the fastest convergence speed compared with the SFOA and other algorithms. Through testing the multimodal function and multimodal function of fixed dimensions, it can be clearly seen that the SFOAL has a stronger global optimization ability and a smaller standard deviation than other algorithms, indicating that the joint strategy can prevent the SFOAL from falling into local convergence too early and can effectively improve stability. The results of the UWB line-of-sight positioning problem show that the SFOAL-BP neural network has the smallest average position error compared with the random BP neural network and the SFOA-BP neural network, and it can be used to solve practical application problems.

In future studies, by mixing the SFOA with other algorithms, it may be possible to further improve the algorithm’s global optimization ability, avoid premature convergence, improve the algorithm’s stability, and use it for solving more functions. The enhanced SFOAL can be applied to artificial intelligence and machine learning. Through reinforcement learning algorithms, different network layers, the number of neurons, the convolution kernel size, and other architectural parameters can be explored to find the neural network architecture with the best performance in specific tasks (such as image classification, speech recognition, etc.), reducing the time and workload required for manually designing network architectures.

## Figures and Tables

**Table 1:** Results of unimodal benchmark functions.

*f*	Results	SFOAL	SFOA	GWO	GOOSE	GA	PKO	DOA	COA	BWO	ESC
*F*_1_	min	0	0	8.73 × 10^−29^	0.0059	4850.2	0.0002	0.0228	0	0.0007	0.0001
	std	0	0	1.75 × 10^−27^	57.47	4627.3	0.0719	0.0256	0	0.0047	7.84 × 10^−05^
	avg	0	0	9.93 × 10^−28^	13.26	12,149.3	0.0682	0.0595	0	0.0060	0.0001
*F*_2_	min	0	0	1.50 × 10^−17^	0.4634	26.05	0.0002	0.0550	7.1 × 10^−192^	0.0086	0.0235
	std	0	0	6.96 × 10^−17^	781.5	9.20	0.0733	0.0142	0	0.0195	0.0081
	avg	0	0	9.44 × 10^−17^	243.5	39.18	0.0325	0.082	4.31 × 10^−183^	0.0317	0.0351
*F*_3_	min	0	0	1.89 × 10^−09^	1868.2	21,483.7	594.72	1679.8	0	0.0743	609.5
	std	0	0	3.06 × 10^−05^	2035.3	9322.8	1241.69	957.8	0	1.99	1168.7
	avg	0	0	1.36 × 10^−05^	4701.8	35,837.1	2181.47	3238.6	0	2.29	2820.6
*F*_4_	min	0	0	3.59 × 10^−08^	0.1472	59.526	2.72	2.50	2.38 × 10^−195^	0.0055	1.13
	std	0	0	8.80 × 10^−07^	21.79	6.08	3.227	0.92	0	0.0058	4.73
	avg	0	0	6.66 × 10^−07^	23.62	74.15	5.96	4.14	1.28 × 10^−179^	0.0147	5.81
*F*_5_	min	1.09 × 10^−07^	28.1595	25.83	25.58	41,406.6	28.20	29.98	1.32 × 10^−06^	0.0010	27.55
	std	0.0002	0.257179	0.7512	154.07	61,2151.4	288.79	45.05	0.4099	0.0182	109.4
	avg	0.0001	28.6312	26.96	121.84	48,6956.2	177.56	108.64	0.1865	0.0243	105.1
*F*_6_	min	2.47 × 10^−07^	0.285645	0.2503	0.0058	3390.22	7.70 × 10^−05^	0.0327	0.0001	0.0289	6.16 × 10^−05^
	std	0.0006	0.3736	0.3650	60.60	6788.07	0.0575	0.0251	0.0104	0.5132	0.0001
	avg	0.0005	0.8034	0.8626	17.66	10,953.75	0.0393	0.0692	0.0081	0.5619	0.0001
*F*_7_	min	1.07 × 10^−05^	1.29 × 10^−05^	0.0007	0.0444	0.1539	0.0079	0.1519	3.30 × 10^−06^	2.23 × 10^−05^	0.0041
	std	0.0001	0.0018	0.0011	0.0573	1.09	0.0262	0.2674	0.0001	0.0003	0.0043
	avg	0.00014	0.0016	0.0020	0.1159	1.07	0.0440	0.4121	0.0001	0.0006	0.0106

**Table 2:** Results of multimodal functions.

*f*	Results	SFOAL	SFOA	GWO	GOOSE	GA	PKO	DOA	COA	BWO	ESC
*F*_8_	min	−12,569	−7380	−7879	−7966	−3092	−7951	−12,332	−12,569	−12,569	−11,868
	std	778.1	856.948	703.4	572.1	588.66	363.33	280.4	0.1864	5.66	425.1
	avg	−12,033	−5800	−6308	−6945	−2115	−6984	−11,900	−12,569	−12,562	−11,146
*F*_9_	min	0	0	5.68 × 10^−14^	87.4	201.0	4.74	1.0307	0	0.0002	10.8
	std	0	0	3.97	37.76	31.24	21.3	1.84	0	0.0018	4.67
	avg	0	0	2.52	165.83	256.2	33.0	4.25	0	0.0019	17.9
*F*_10_	min	4.44 × 10^−16^	4.44 × 10^−16^	7.50 × 10^−14^	0.0972	19.4	0.0018	0.0479	4.44 × 10^−16^	0.0015	0.0064
	std	0	0	2.11 × 10^−14^	9.08	0.3305	10.0	0.0135	0	0.0102	0.8686
	avg	4.44 × 10^−16^	4.44 × 10^−16^	1.06 × 10^−13^	12.64	20.1	11.9	0.0733	4.44−16	0.0173	1.04
*F*_11_	min	0	0	0	0.0005	27.7	4.47 × 10^−05^	0.0626	0	0.0017	1.35 × 10^−05^
	std	0	0	0.0074	209.0	62.8	0.0902	0.0386	0	0.0155	0.1716
	avg	0	0	0.0038	274.8	114.3	0.0652	0.1302	0	0.0165	0.1161
*F*_12_	min	1.25 × 10^−07^	0.0081	0.0129	5.33	1.57	8.72 × 10^−05^	8.02 × 10^−05^	1.28 × 10^−07^	5.87 × 10^−05^	1.08 × 10^−06^
	std	1.21 × 10^−05^	0.01855	0.1192	5.65	4.82	2.66	0.0463	0.0001	0.0005	0.1026
	avg	1.06 × 10^−05^	0.0306	0.0687	15.79	9.97	1.47	0.0105	9.99 × 10^−05^	0.0007	0.0366
*F*_13_	min	1.39 × 10^−07^	0.3418	0.2993	0.0021	39.3	0.0016	0.0011	6.22 × 10^−06^	1.95 × 10^−05^	1.19 × 10^−05^
	std	0.0001	0.6050	0.2130	10.71	969,207	15.1	0.0041	0.0033	0.0001	0.6936
	avg	0.000105	0.9900	0.583909	5.46	548,605	7.22	0.0035	0.0012	0.0001	0.3416

**Table 3:** Results of multimodal functions with fixed variables (*F*_14_–*F*_18_).

*f*	Results	SFOAL	SFOA	GWO	GOOSE	GA	PKO	DOA	COA	BWO	ESC
*F*_14_	min	0.998	0.998	0.998	1.99	0.998	0.998	0.998	0.998	0.998	0.998
	std	2.48−15	1.64 × 10^−14^	5.05	5.46	3.33 × 10^−10^	0	2.62 × 10^−14^	2.19−10	2.055	5.09 × 10^−17^
	avg	0.9980	0.998	5.78	11.5	0.998	0.998	0.998	0.9980	5.06	0.998
*F*_15_	min	0.0003	0.0003	0.0003	0.0003	0.0008	0.0005	0.0003	0.0003	0.0003	0.0003
	std	2.33−10	0.0002	0.0002	0.0151	0.0137	0.0002	0.0043	0.0002	5.03 × 10^−05^	0.0001
	avg	0.0003	0.0003	0.0004	0.0075	0.0142	0.0009	0.0019	0.0005	0.0004	0.0007
*F*_16_	min	−1.03	−1.03	−1.03	−1.03	−1.03	−1.03	−1.03	−1.03	−1.03	−1.03
	std	7.41 × 10^−09^	9.74 × 10^−11^	1.48−08	0.2989	0.114	2.27 × 10^−16^	2.86−10	3.80 × 10^−06^	0.0003	1.76 × 10^−16^
	avg	−1.03	−1.03	−1.03	−0.9092	−0.9720	−1.03	−1.03	−1.03	−1.03	−1.031
*F*_17_	min	0.3978	0.3978	0.3978	0.3978	0.3978	0.3978	0.3978	0.3978	0.3982	0.3978
	std	7.79 × 10^−08^	5.13 × 10^−06^	3.37 × 10^−06^	3.07 × 10^−10^	7.72 × 10^−05^	0	0.0001	3.10 × 10^−10^	0.0060	0
	avg	0.3978	0.3978	0.3978	0.3978	0.3979	0.3978	0.3979	0.3978	0.4041	0.3978
*F*_18_	min	3	3	3	3	3	3	3	3	3.25	3
	std	4.18 × 10^−13^	2.43 × 10^−15^	18.1	25.19915417	0.0004	7.13 × 10^−16^	6.03	0.0012	11.1	4.66 × 10^−16^
	avg	3	3	7.05	12.4	3	3	4.35	3	18.7	3

**Table 4:** Statistical results of multimodal functions with fixed variables (*F*_19_–*F*_23_).

*f*	Results	SFOAL	SFOA	GWO	GOOSE	GA	PKO	DOA	COA	BWO	ESC
*F*_19_	min	−3.86	−3.86	−3.86	−3.86	−3.83	−3.86	−3.86	−3.86	−3.80	−3.86
	std	2.39 × 10^−10^	2.30 × 10^−15^	0.0021	1.04 × 10^−06^	0.4353	2.21 × 10^−15^	0.0001	0.0182	0.0682	2.27 × 10^−15^
	avg	−3.86	−3.86	−3.86	−3.86	−3.3	−3.86	−3.86	−3.85	−3.71	−3.86
*F*_20_	min	−3.32	−3.32	−3.32	−3.32	−2.41	−3.32	−3.32	−3.2	−3.15	−3.32
	std	0.0265	0.0481	0.0710	0.0606	0.4605	0.0487	0.0014	0.3949	0.4079	0.0581
	avg	−3.31	−3.29	−3.26	−3.24	−1.69	−3.29	−3.31	−2.72	−2.75	−3.28
*F*_21_	min	−10.15	−10.15	−10.15	−10.15	−2.73	−10.15	−10.07	−10.15	−10.15	−10.15
	std	3.69 × 10^−07^	1.12	1.56	2.26	0.5036	2.09	2.37	0.0001	0.0162	3.39
	avg	−10.15	−9.90	−9.64	−4.50	−1.57	−9.13	−7.51	−10.15	−10.13	−7.23
*F*_22_	min	−10.40	−10.40	−10.40	−10.40	−3.88	−10.40	−10.33	−10.40	−10.39	−10.40
	std	2.98 × 10^−07^	4.08 × 10^−06^	1.70	2.70	0.9440	2.18	1.99	0.0001	0.0233	1.51
	avg	−10.40	−10.40	−10.01	−4.57	−1.87	−9.33	−8.89	−10.40	−10.37	−9.99
*F*_23_	min	−10.53	−10.53	−10.53	−10.53	−3.8	−10.53	−10.51	−10.53	−10.53	−10.53
	std	7.06 × 10^−06^	0.0002	0.0010	3.60	0.7117	1.20	2.43	8.45 × 10^−05^	0.0155	2.59
	avg	−10.53	−10.53	−10.53	−4.48	−1.88	−10.26	−8.59	−10.53	−10.51	−9.27

**Table 5:** Wilcoxon rank sum test results.

*f*	SFOA	GWO	GOOSE	GA	PKO	DOA	COA	BWO	ESC
*F*_1_	1	8.00 × 10^−09^	8.00 × 10^−09^	8.00 × 10^−09^	8.00 × 10^−09^	8.00 × 10^−09^	1	8.00 × 10^−09^	8.00 × 10^−09^
*F*_2_	1	8.00 × 10^−09^	8.00−09	8.00 × 10^−09^	8.00 × 10^−09^	8.00 × 10^−09^	8.00 × 10^−09^	8.00 × 10^−09^	8.00 × 10^−09^
*F*_3_	1	8.00 × 10^−09^	8.00 × 10^−09^	8.00 × 10^−09^	8.00 × 10^−09^	8.00 × 10^−09^	1	8.00 × 10^−09^	8.00 × 10^−09^
*F*_4_	1	8.00 × 10^−09^	8.00−09	8.00 × 10^−09^	8.00 × 10^−09^	8.00 × 10^−09^	8.00 × 10^−09^	8.00 × 10^−09^	8.00 × 10^−09^
*F*_5_	6.79 × 10^−08^	6.79 × 10^−08^	6.79 × 10^−08^	6.79 × 10^−08^	6.79 × 10^−08^	6.79 × 10^−08^	2.35 × 10^−06^	6.79 × 10^−08^	6.79 × 10^−08^
*F*_6_	6.79 × 10^−08^	6.79 × 10^−08^	6.79−08	6.79 × 10^−08^	0.0123	6.79 × 10^−08^	0.0001	6.79 × 10^−08^	0.0531
*F*_7_	0.0001	6.79 × 10^−08^	6.79 × 10^−08^	6.79 × 10^−08^	6.7 × 10^−08^	6.79 × 10^−08^	0.9245	5.89 × 10^−05^	6.7 × 10^−08^
*F*_8_	6.79 × 10^−08^	6.79 × 10^−08^	6.79 × 10^−08^	6.79 × 10^−08^	6.79 × 10^−08^	0.0810	4.53 × 10^−07^	1	0.0001
*F*_9_	1	7.71 × 10^−09^	8.00 × 10^−09^	8.00 × 10^−09^	8.00 × 10^−09^	8.00 × 10^−09^	1	8.00 × 10^−09^	8.00 × 10^−09^
*F*_10_	1	7.78 × 10^−09^	8.00 × 10^−09^	8.00 × 10^−09^	8.00 × 10^−09^	8.00 × 10^−09^	1	8.00 × 10^−09^	8.00 × 10^−09^
*F*_11_	1	0.0095	8.00 × 10^−09^	8.00 × 10^−09^	8.00 × 10^−09^	8.00 × 10^−09^	1	8.00 × 10^−09^	8.00 × 10^−09^
*F*_12_	6.79−08	6.79 × 10^−08^	6.79 × 10^−08^	6.79 × 10^−08^	6.79 × 10^−08^	6.79 × 10^−08^	0.0207	6.79 × 10^−08^	0.1895
*F*_13_	6.79 × 10^−08^	6.79 × 10^−08^	6.79 × 10^−08^	6.79 × 10^−08^	6.79 × 10^−08^	6.79 × 10^−08^	0.0027	0.0256	0.0001
*F*_14_	0.5816	6.10× 10^−08^	6.01 × 10^−08^	6.19 × 10^−08^	6.97 × 10^−09^	4.40 × 10^−07^	6.71 × 10^−06^	6.10× 10^−08^	9.86 × 10^−09^
*F*_15_	0.0005	6.79−08	6.79 × 10^−08^	6.79 × 10^−08^	6.7 × 10^−08^	6.79 × 10^−08^	6.79 × 10^−08^	6.79 × 10^−08^	6.79 × 10^−08^
*F*_16_	0.1328	3.06 × 10^−06^	0.0016	6.79 × 10^−08^	8.0 × 10^−09^	0.0097	2.92 × 10^−05^	6.79−08	3.95 × 10^−08^
*F*_17_	0.0030	2.21 × 10^−07^	1.20 × 10^−06^	6.79 × 10^−08^	2.99 × 10^−08^	1.91 × 10^−07^	5.25 × 10^−05^	6.79 × 10^−08^	2.99 × 10^−08^
*F*_18_	6.43 × 10^−08^	6.79 × 10^−08^	6.79 × 10^−08^	6.79 × 10^−08^	2.93 × 10^−08^	6.79 × 10^−08^	6.79 × 10^−08^	6.79 × 10^−08^	3.93 × 10^−08^
*F*_19_	4.45 × 10^−08^	6.79−08	6.79 × 10^−08^	6.79 × 10^−08^	1.94 × 10^−08^	6.79 × 10^−08^	6.79 × 10^−08^	6.79−08	8.00 × 10^−09^
*F*_20_	0.2284	3.41 × 10^−07^	2.2 × 10^−07^	6.79 × 10^−08^	0.0007	1.20 × 10^−06^	9.17 × 10^−08^	6.79 × 10^−08^	0.1257
*F*_21_	0.4569	6.79 × 10^−08^	6.7 × 10^−08^	6.79 × 10^−08^	0.001	6.79 × 10^−08^	3.70 × 10^−05^	6.79 × 10^−08^	0.5963
*F*_22_	0.7971	6.79−08	7.8 × 10^−08^	6.79 × 10^−08^	0.0009	6.79 × 10^−08^	6.79 × 10^−08^	6.79−08	6.03 × 10^−06^
*F*_23_	0.3507	6.79 × 10^−08^	1.06 × 10^−07^	6.79 × 10^−08^	3.53 × 10^−07^	6.79 × 10^−08^	1.06−07	6.79 × 10^−08^	0.0011

**Table 6:** Results of CEC2021.

*f*	Results	SFOAL	SFOA	GWO	GOOSE	GA	PKO	DOA	COA	BWO	ESC
*f*_1_	min	0	0	7.59 × 10^−32^	912.9	12,604,415	0.0054	3679.3	0	82.62	4.78
	std	0	0	1.00 × 10^−28^	1612.1	124,214,368	601.3	1765.3	0	4562.0	516.6
	avg	0	0	3.40 × 10^−29^	2474.0	80,788,533	408.1	4956.4	0	2851.3	303.7
*f*_2_	min	0	0	1.8 × 10^−12^	0.0166	3264.9	326.9	1.89	0	0.0033	1.93
	std	0	0	10.22	1707.1	488.2	513.0	37.70	0	0.0143	13.42
	avg	0	0	9.01	2103.4	4110.1	1087.1	18.68	0	0.0221	16.33
*f*_3_	min	0	0	2.40 × 10^−29^	18.91	105.2	6.60	2.06	0	0.0021	23.57
	std	0	0	53.26	645.7	51.1	28.35	9.17	0	0.0110	4.95
	avg	0	0	88.19	351.0	180.2	37.54	13.82	0	0.0139	29.05
*f*_4_	min	0	0	0	1.26	4.33	0.0653	0.7538	0	3.10 × 10^−09^	2.26
	std	0	0	0.4398	31.63	1.48	1.86	0.3659	0	2.13 × 10^−08^	1.12
	avg	0	0	0.2535	46.70	7.26	3.85	1.34	0	2.54 × 10^−08^	3.52
*f*_5_	min	0	0	7.38 × 10^−17^	1549.1	8115.2	23.75	0.4179	0	15.26	2.23
	std	0	0	13.14	170,910	172,131	70.24	3.73	0	428.21	97.95
	avg	0	0	11.09	145,504	115,801	77.57	2.64	1.00 × 10^−279^	327.3	90.73
*f*_6_	min	00	0	0.0832	0.9589	29.93	4.31	0.4496	−1.11 × 10^−16^	0.0520	1.01
	std	0	0	26.49	666.9	159.0	58.49	2.90	3.51 × 10^−17^	0.0580	3.80
	avg	0	0	12.46	671.5	194.8	45.74	3.10	−1.11 × 10^−17^	0.1037	6.82
*f*_7_	min	0	0	0.0631	452.5	1550.9	1.57	1.15	−1.11 × 10^−16^	24.00	1.06
	std	0	0	3.87	22,544	70,050	39.71	0.7521	4.68 × 10^−17^	105.9	40.04
	avg	0	0	2.21	12,521	64,965	21.61	1.92	−2.22 × 10^−17^	150.8	17.31
*f*_8_	min	0	0	0	0.0291	424.6	2.92 × 10^−06^	0.0438	0	0.0032	9.88
	std	0	0	0	1934.1	1317.5	16.32	4.6	0	0.0506	23.65
	avg	0	0	0	2233.9	2861.6	14.24	2.28	0	0.0824	29.55
*f*_9_	min	0	0	3.55 × 10^−14^	0.0757	71.31	0.0001	0.172587767	0	0.0535	0.003
	std	0	0	7.48 × 10^−15^	64.35	42.26	0.0068	0.074036009	0	0.0943	0.0037
	avg	0	0	4.97 × 10^−14^	31.39	122.50	0.0053	0.259506615	0	0.1672	0.0078
*f*_10_	min	0	0	52.78	0.1264	70.82	49.91	49.05334189	0	0.1357	48.85
	std	0	0	12.53	54.51	22.93	2.02	8.680866255	0	0.1018	16.79
	avg	0	0	77.83	107.1	101.87	51.56	53.06522585	0	0.2500	62.39

## Data Availability

All relevant data are within the paper.

## Appendix A

**Table A1:** Unimodal functions.

Benchmark Functions	Range	*f*_min_
$F_{1}(x)=\sum_{i=1}^{D}x_{i}^{2}$	[−100,100]	0
$F_{2}(x)=\sum_{i=1}^{D}\left|x_{i}\right|+\prod_{i=1}^{D}\left|x_{i}\right|$	[−10,10]	0
$F_{3}(x)={\sum_{i=1}^{D}\left(\sum_{j=1}^{i}x_{j}\right)}^{2}$	[−100,100]	0
$F_{4}(x)=\max_{i}\{\left|x_{i}\right|\}$	[−100,100]	0
$F_{5}(x)=\sum_{i=1}^{D-1}\left[100{\left(x_{i+1}-x_{i}^{2}\right)}^{2}+{\left(x_{i}-1\right)}^{2}\right]$	[−30,30]	0
$F_{6}(x)={\sum_{i=1}^{D}\left(\left\lfloor x_{i}+0.5\right\rfloor \right)}^{2}$	[−100,100]	0
$F_{7}(x)=\sum_{i=1}^{D}ix_{i}^{2}+\mathrm{random}[0,1)$	[−1.28,1.28]	0

**Table A2:** Multimodal functions.

Benchmark Functions	Range	*f*_min_
$F_{8}(x)=\sum_{i=1}^{D}x_{i}^{2}\sin(\sqrt{\left|x_{i}\right|})$	[−500,500]	−418.9829D
$F_{9}(x)=\sum_{i=1}^{D}[x_{i}^{2}-10\cos(2\pi x_{i})+10]$	[−5.12,5.12]	0
$\begin{array}{l} F_{10}(x)=-20\exp(-0.2\sqrt{\frac{1}{D}\sum_{i=1}^{D}x_{i}^{2}}-\exp(\frac{1}{D}\sum_{i=1}^{D}\cos(2\pi x_{i}))\\ +20+e \end{array}$	[−32,32]	0
$F_{11}(x)=\sum_{i=1}^{D}\frac{{x_{i}}^{2}}{4000}-\prod_{i=1}^{D}\cos(\frac{x_{i}}{\sqrt{i}})+1$	[−600,600]	0
$\begin{array}{l} F_{12}(x)=\frac{\pi }{D}\{10\sin^{2}(\pi y_{1})+\sum_{i=1}^{D-1}{\left(y_{i}-1\right)}^{2}[1+10\sin^{2}(\pi y_{i+1})]\\ +{\left(y_{D}-1\right)}^{2}\}+\sum_{i=1}^{D}\mu (x_{i},10,100,4)\\ y_{i}=1+\frac{x_{i}+1}{4}\\ \mu (x_{i},a,k,m)=\left\{\begin{array}{ccc} k{\left(x_{i}-a\right)}^{m} & & x_{i}>a\\ 0 & & -a\leq x_{i}\leq a\\ k{\left(-x_{i}-a\right)}^{m} & & x_{i}<-a \end{array}\right. \end{array}$	[−50,50]	0
$\begin{array}{l} F_{13}(x)=0.1(\sin2(3\pi x1+g(x)))+\sum_{i=1}^{D}\mu (x_{i},5,100,4)\\ g(x)=\sum_{i=1}^{D-1}{\left(x_{i}-1\right)}^{2}[1+\sin^{2}(3\pi x_{i}+1)]\\ +{\left(x_{D}-1\right)}^{2}[1+\sin^{2}(2\pi x_{D})] \end{array}$	[−50,50]	0

**Table A3:** Fixed-dimension multimodal functions.

Benchmark Functions	Range	*f*_min_
$F_{14}(x)={\left(\frac{1}{500}+\sum_{j=1}^{25}\frac{1}{j+\sum_{i=1}^{2}{\left(x_{i}-a_{ij}\right)}^{6}}\right)}^{-1}$	[−65,65]	1
$F_{15}(x)={\sum_{i=1}^{11}\left[a_{i}-\frac{x_{1}(b_{i}^{2}+b_{1}x_{2})}{b_{i}^{2}+b_{1}x_{3}+x_{4}}\right]}^{2}$	[−5,5]	0.1484
$F_{16}(x)=4x_{1}^{2}-2.1x_{1}^{4}+\frac{1}{3}x_{1}^{6}+x_{1}x_{2}-4x_{2}^{2}+4x_{2}^{4}$	[−5,5]	−1.0316
$F_{17}(x)={\left(x_{2}-\frac{5.1}{4\pi^{2}}x_{1}^{2}+\frac{5}{\pi }x_{1}-6\right)}^{2}+10\left(1-\frac{1}{8\pi }\right)\cos x_{1}+10$	[−5,5], [10,15]	0.3979
$\begin{array}{l} F_{18}(x)=[1+{\left(x_{1}+x_{2}+1\right)}^{2}(19-14x_{1}+3x_{1}^{2}-14x_{2}+6x_{1}x_{2}+3x_{2}^{2}]\\ \times [30+{\left(2x_{1}-3x_{2}\right)}^{2}(18-32x_{1}+12x_{1}^{2}+48x_{2}-36x_{1}x_{2}+27x_{2}^{2})] \end{array}$	[−2,2]	3
$F_{19}(x)=-\sum_{i=1}^{4}c_{i}\exp\left(-\sum_{j=1}^{3}a_{ij}{\left(x_{j}-pi_{j}\right)}^{2}\right)$	[0,1]	−3
$F_{20}(x)=-\sum_{i=1}^{4}c_{i}\exp\left(-\sum_{j=1}^{36}a_{ij}{\left(x_{j}-pi_{j}\right)}^{2}\right)$	[0,1]	−3
$F_{21}(x)=-{\sum_{i=1}^{5}\left[(X-a_{i}){\left(X-a_{i}\right)}^{T}+c_{i}\right]}^{-1}$	[0,10]	−1
$F_{22}(x)=-{\sum_{i=1}^{7}\left[(X-a_{i}){\left(X-a_{i}\right)}^{T}+c_{i}\right]}^{-1}$	[0,10]	−1
$F_{23}(x)=-{\sum_{i=1}^{10}\left[(X-a_{i}){\left(X-a_{i}\right)}^{T}+c_{i}\right]}^{-1}$	[0,10]	−1
